# Effects of a patient-centered digital health intervention in patients referred to cardiac rehabilitation: the Smart HEART clinical trial

**DOI:** 10.1186/s12872-023-03471-w

**Published:** 2023-09-12

**Authors:** Arash Harzand, Alaaeddin Alrohaibani, Muhammed Y. Idris, Hayden Spence, Cate G. Parrish, Pratik K. Rout, Rene Nazar, Michelle L. Davis-Watts, Phyllis P. Wright, Alexander A. Vakili, Smah Abdelhamid, Harshvardhan Vathsangam, Adelanwa Adesanya, Linda G. Park, Mary A. Whooley, Nanette K. Wenger, A. Maziar Zafari, Amit J. Shah

**Affiliations:** 1 Atlanta VA Medical Center, 1670 Clairmont Road (111/CD), Decatur, GA 30033 USA; 2 Division of Cardiology, School of Medicine, Emory University, Atlanta, GA USA; 3 Department of Pathology, Oregon Health and Science University, Portland, OR USA; 4 Department of Medicine, Morehouse School of Medicine, Atlanta, GA USA; 5 Aptive Resources, LLC, Alexandria, VA USA; 6 Movn Health, Irvine, CA USA; 7 School of Nursing, University of California, San Francisco, CA USA; 8 San Francisco VA Medical Center, San Francisco, CA USA; 9 Department of Medicine, University of California, San Francisco, CA USA; 10 Department of Epidemiology, Emory University Rollins School of Public Health, Atlanta, GA USA

**Keywords:** Digital technology, Cardiac rehabilitation, Exercise therapy patient-centered care, Pragmatic clinical trial

## Abstract

**Background:**

Cardiac rehabilitation (CR) improves outcomes in heart disease yet remains vastly underutilized. Remote CR enhanced with a digital health intervention (DHI) may offer higher access and improved patient-centered outcomes over non-technology approaches. We sought to pragmatically determine whether offering a DHI improves CR access, cardiac risk profile, and patient-reported outcome measures.

**Methods:**

Adults referred to CR at a tertiary VA medical center between October 2017 and December 2021 were offered enrollment into a DHI alongside other CR modalities using shared decision-making. The DHI consisted of remote CR with a structured, 3-month home exercise program enhanced with multi-component coaching, a commercial smartphone app, and wearable activity tracker. We measured completion rates among DHI participants and evaluated changes in 6-min walk distance, cardiovascular risk factors, and patient-reported outcomes from pre- to post-intervention.

**Results:**

Among 1,643 patients referred to CR, 258 (16%) consented to the DHI where the mean age was 60 ± 9 years, 93% were male, and 48% were black. A majority (90%) of the DHI group completed the program. Over 3-months, significant improvements were seen in 6MWT (mean difference [MD] -29 m; 95% CI, 10 to 49; *P* < 0.01) and low-density lipoprotein cholesterol (MD -11 mg/dL; 95% CI, -17 to -5; *P* < 0.01), and the absolute proportion of patients who reported smoking decreased (10% vs 15%; MD, -5%; 95% CI, -8% to -2%; *P* < 0.01) among DHI participants with available data. No adverse events were reported.

**Conclusions:**

The addition of a DHI-enhanced remote CR program was delivered in 16% of referred veterans and associated with improved CR access, markers of cardiovascular risk, and healthy behaviors in this real-world study. These findings support the continued implementation of DHIs for remote CR in real-world clinical settings.

**Trial registration:**

This trial was registered on ClinicalTrials.gov: NCT02791685 (07/06/2016).

## Background

Cardiac rehabilitation (CR) has been shown to reduce hospital readmissions, recurrent cardiovascular events, depression, and mortality, yet overall participation remains low [1]. Fewer than one-third of eligible US adults participate, mainly due to limited availability and inconvenience [2, 3]. Further disparities have been seen in specific high-risk populations, including racial minorities, older adults, and military veterans [4–7]. New CR delivery strategies that can be easily adapted to rapidly shifting patient- and system-level factors are needed to expand access to guideline-directed CR programs, particularly among historically vulnerable groups [8].

Digital health interventions (DHI) that promote self-management can effectively deliver patient-centered cardiovascular prevention programs [9–13]. When used for remote CR, DHIs lead to improved control of cardiac risk factors, improved functional capacity, and high patient satisfaction [14–17]. Prior interventions have had limited real-world evidence on patient engagement or health outcomes from routine clinical settings, however [18]. It also remains unclear if DHIs for remote CR can be successfully deployed among high-risk populations where mobile adoption and broadband access remain limited [19]. This question is of particular relevance to veterans, of whom many are older, low-income, and live in rural areas with poor internet access [20]. These factors further reduce the real-world feasibility of emerging care technologies.

Smart HEART (Health Education and Rehabilitation Technology) is a comprehensive DHI for remote CR delivery that integrates mobile health technologies into home-based exercise training and lifestyle counseling. We previously reported the feasibility of Smart HEART in veterans demonstrating high engagement and patient satisfaction scores, [21] and have since integrated the program into our CR workflow such that each patient referred to CR is routinely offered the program. The program has several advantages, such as providing direct communication between the patient and a coach, a mobile smartphone app to guide program delivery and encourage self-monitoring, and a wrist-worn activity tracker to encourage regular exercise.

In this study, we describe the real-world impact of Smart HEART following implementation within a single Department of Veterans Affairs (VA) medical center. We used a pragmatic, open-label study design as a resource-efficient method to measure the impact of Smart HEART on CR access, measures of cardiovascular health, and patient-reported outcomes within routine clinical practice.

## Methods

### Study overview & design

The current analysis focuses on unique patients referred for CR between October 2017 and December 2021 at the Atlanta VA Medical Center, part of the Veterans Health Administration (VHA). Using a shared decision-making model that considered each patient’s preference, cardiac risk profile, and technology literacy, sequentially referred patients were offered enrollment into our facility’s remote CR program (either with or without the DHI-enhancements) in addition to enrollment into a traditional, center-based CR program at a non-VA facility through the VA Community Care Program. Enrollment in remote and center-based CR was not considered mutually exclusive to encourage patient choice, and patients were permitted to participate in both if they desired. We present data from the DHI program only, as data from the other CR programs were severely limited and outside of the scope of this study.

The DHI components were developed by an industry partner (Movn Health, Irvine, California) who was not involved in the study design but participated in training the study team and delivering parts of the DHI intervention, including direct patient coaching and data collection. All the patients provided informed consent before enrollment. The Institutional Review Board at Emory University and the Research & Development Committee at the Department of Veterans Affairs approved the study protocol. The study was registered with the U.S. National Library of Medicine ClinicalTrials.gov registry (NCT02791685) on 07/06/2016.

### Participants

Eligible patients were all adults aged 21 years or older referred to CR with a qualifying diagnosis per established criteria (e.g., acute myocardial infarction, percutaneous coronary intervention, coronary artery bypass grafting, heart valve surgery, chronic stable angina pectoris, or chronic systolic heart failure) [22]. Patients with active or unstable cardiac conditions, acute systemic illness, high-risk findings on pre-enrollment exercise testing, or an inability to exercise due to a non-cardiac condition were excluded. Patients were required to own an Android or an iOS smartphone in working condition with access to Wi-Fi or a data plan to enroll.

### Digital health intervention

**Fig. 1:**
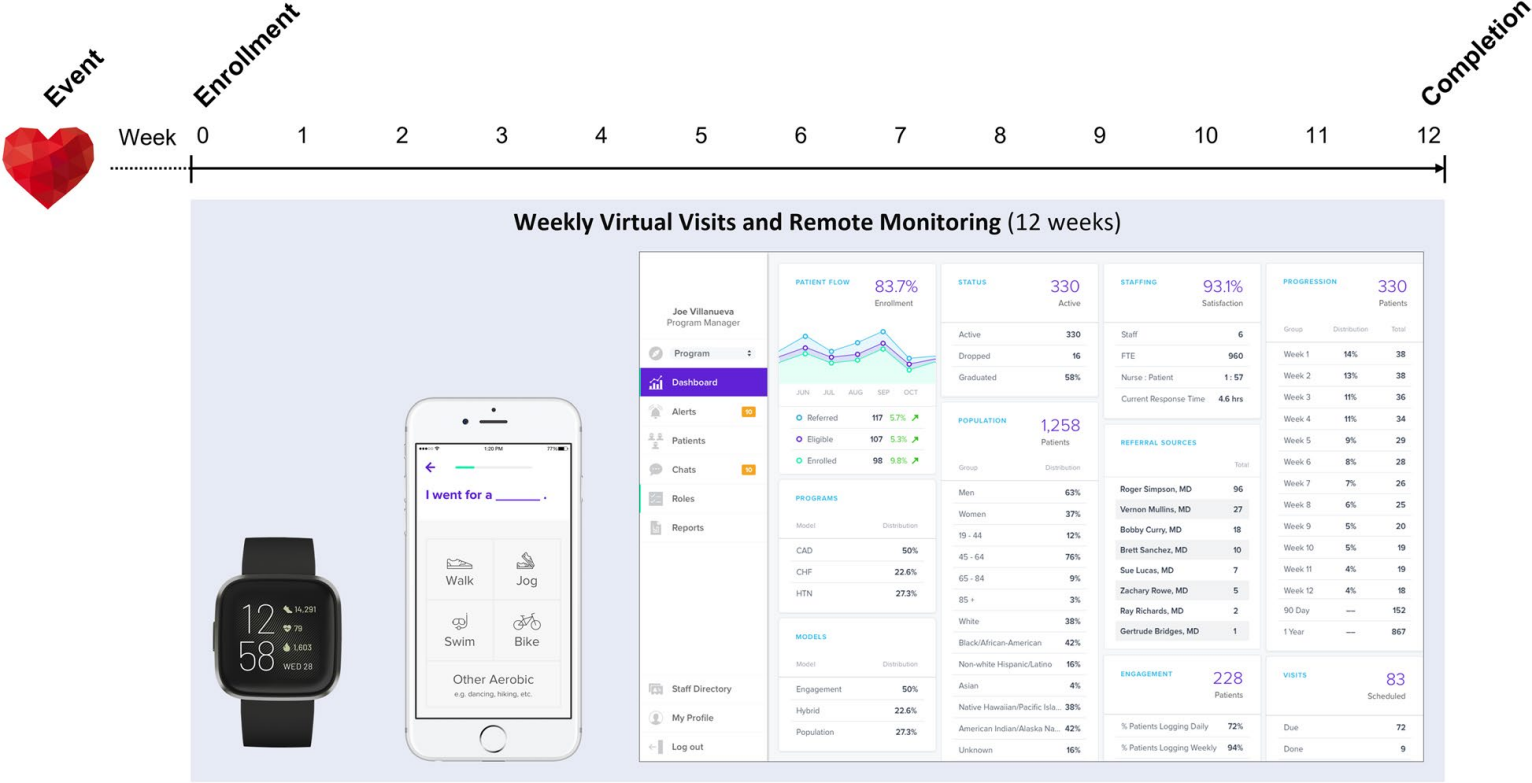
Movn digital health intervention which includes a wrist-born activity tracker, patient-facing smartphone app, and provider management dashboard. Note that demographic and other numerical data in the figure are representative only and do not reflect results from the current study

Movn is a commercially available program for remote CR based on MULTIFIT, [23, 24] a case-management system for secondary prevention and patient surveillance after acute MI initially developed by investigators at Stanford University School of Medicine and broadly implemented across Kaiser Permanente [24]. Following an in-person or remote baseline visit, participants in the DHI received a 3-month, remote CR program consisting of structured home exercise enhanced with the Movn smartphone app and a wearable fitness tracker (Fitbit Charge HR, Garmin vivosmart, or similar device) to self-monitor exercise activity and share data with a dedicated health coach (Fig. 1). Participants received remote monitoring and weekly phone or telehealth-based visits from the coach (an advanced practice provider or exercise physiologist), who provided structured education on risk reduction and adopting healthy habits and made referrals as needed to ancillary services, including nutrition and health psychology.

The coach developed an exercise prescription tailored for each participant based on a target range for HR derived from a pre-enrollment 6-min walk test (6MWT) or using the rate of perceived exertion (RPE) based on the Borg scale [25] if baseline exercise testing data was not available. If necessary, the coach was able to modify a participant’s exercise plan as warranted in response to each participant’s progress.

DHI participants then received reminders to exercise via the Movn app, where they could also self-log exercise activity, vital signs, and medication adherence. The coach could passively monitor each participant’s data through an integrated online case-management dashboard provided by Movn. They could also send health-related surveys to each participant at the beginning of the program, each week, or at the completion of the DHI.

### Study outcomes and clinical endpoints

The primary outcome was the overall rate of DHI enrollment and completion as a proportion of total CR referrals. Secondary outcomes included changes in clinical and patient-reported endpoints following successful completion of the 12-week DHI program, including the 6MWT; [26]; cardiovascular risk factors (e.g., systolic and diastolic blood pressure [BP], [LDL-C], glycosylated hemoglobin A1c, smoking status, and body mass index [BMI]); patient-reported outcome measures (PROMs) including the Duke Activity Status Index (DASI) [27] for functional status and the Patient Health Questionnaire (PHQ-9) [28] for depressive symptoms; and weekly ambulatory activity, mobile app usage, and engagement with the coaching program. All the secondary outcomes were collected by the health coach who was a member of the study team. Patients were asked to complete 6MWT and all survey instruments at entry (i.e., study enrollment) and exit of the 12-week DHI. Surveys were self-completed by the participants.

We defined DHI completion as either 1) participation in a minimum of six (6) remote coaching visits (either telephone or telehealth-based) that were documented as care encounters in the medical record or 2) successful completion of a pre-planned exit visit for follow-up testing. Blinding was not considered feasible based on the nature of the intervention, which required active coordination between study team members and the industry collaborator.

To measure DHI engagement, we calculated the total number of participants and the average number of usage days for various features of the Movn app that required active engagement by the patient, including logging of exercise sessions; self-measured BP, HR, weight, and medication adherence; and self-reported smoking status.

### Statistical methods

Frequencies and percentages were used to summarize all categorical variables, including the primary outcome of DHI completion. Continuous variables were summarized with group means and standard deviations. For within-group comparisons of secondary outcomes, we performed paired t-tests and calculated 95% confidence intervals for all normally distributed data and Wilcoxon rank-sum for highly skewed data. Categorical data were compared using chi-square tests. Secondary outcomes were analyzed based on availability (including via chart review) and not DHI completion. The a priori level for statistical significance was a 2-sided *p* < 0.05. Analyses were performed using SAS Enterprise (Version 9.4; SAS Institute Inc, Cary, NC) and Python (Version 3.10.6; Python Software Foundation, Beaverton, OR).

## Results

### Participant enrollment and completion

**Fig. 2:**
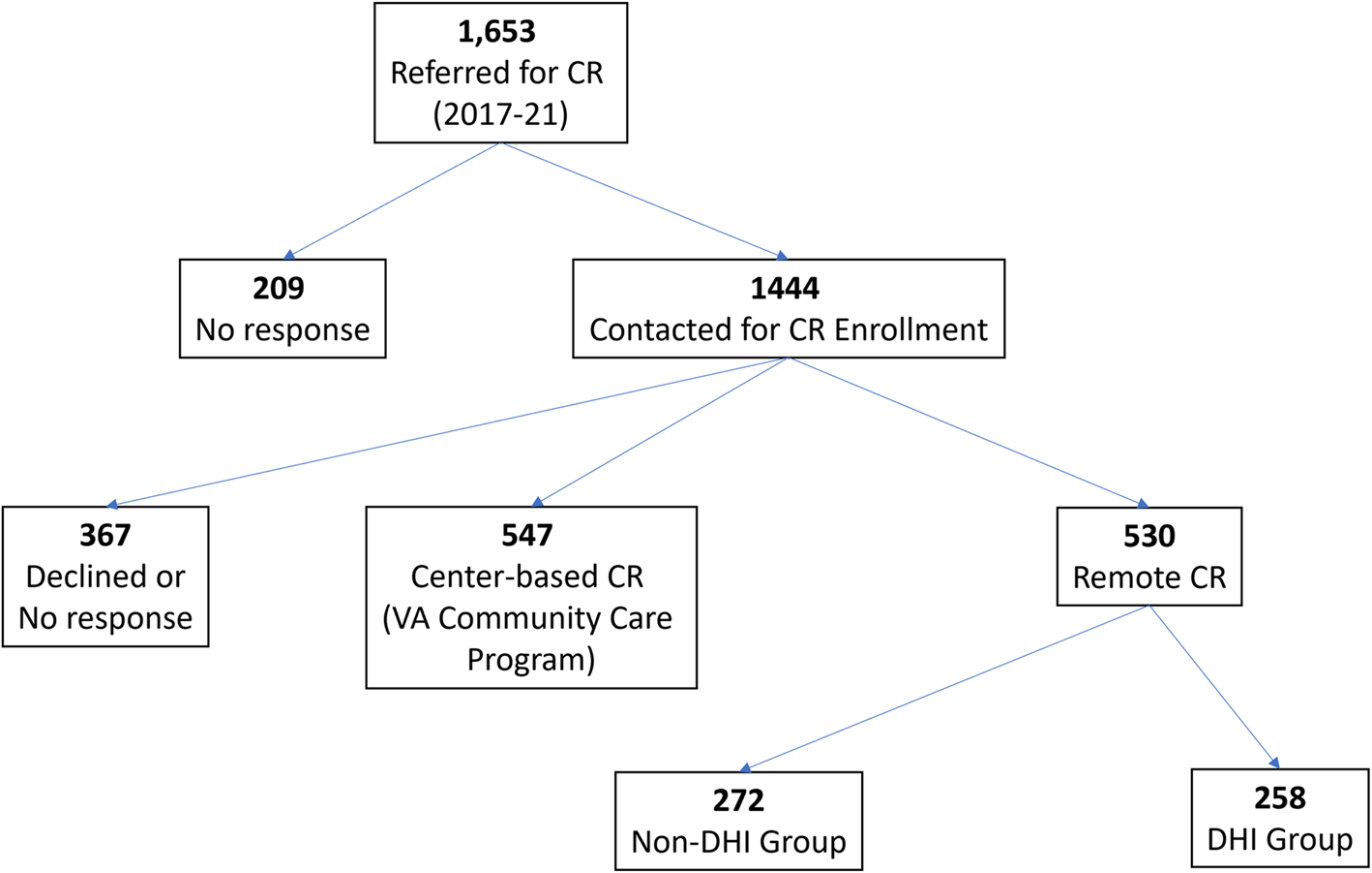
Patient flow diagram

Our facility received 1,653 unique, first-time CR referrals between October 2017 and December 2021 (Fig. 2). Of these, 258 (16%) participants agreed to enroll in remote CR enhanced with the DHI. The remaining patients either participated in traditional center-based CR outside the VA, participated in remote CR without the DHI, or did not successfully enroll in any form of CR. Baseline characteristics for the consented DHI population are provided in Table 1. The mean age for the DHI group was 60 ± 9 years, 93% were male, and 46% were black. A majority (90%) of the enrolled DHI participants completed the entire remote CR program (Table 2).

**Table 1:** Baseline characteristics of participants enrolled in the Movn DHI (*N* = 258)

	**DHI Group** Mean ± SD or n (%)
**Demographics**	
Age, years	60 ± 9
Male	241 (93)
Urban	244 (95)
Rural or very rural	14 (5)
**Race**	
Black^a^	125 (48)
Non-Hispanic White^a^	115 (45)
Hispanic	2 (1)
Other	17
**CR referral indication**	
PCI	110 (43)
Acute MI	41 (16)
CABG	34 (13)
Heart failure	37 (14)
Stable angina	25 (10)
Valve surgery	4 (1)
Other	7 (3)
**History**	
Hypertension	204 (79)
Hyperlipidemia	180 (70)
Coronary artery disease	166 (64)
Diabetes mellitus	98 (38)
Heart failure	61 (24)
Atrial fibrillation	2 (1)
Stroke	12 (5)
Peripheral artery disease	13 (5)
Current smoker	46 (18)
Prior smoker	107 (42)
**Comorbidity Count**	
< 3	90 (35)
3–5	161 (62)
> 5	7 (3)

^a^One patient identified as both black and non-Hispanic white

**Table 2:** Overview of patient enrollment into CR, remote CR, and the DHI over time

**Group**	**Participants per Year** N or N (%)					
	**2017**	**2018**	**2019**	**2020**	**2021**	**Total**
Unique CR referrals^a^	238	338	370	330	377	1653
Enrolled in remote CR (with DHI) DHI^b^	13 (41)	42 (48)	60 (60)	87 (64)	56 (32)	258 (49)
Completed DHI^c^	11 (85)	40 (95)	56 (93)	72 (83)	52 (93)	231 (90)

^a^Total number of unique patients referred to CR by a healthcare provider (repeat referrals excluded)

^b^Total number of remote CR patients accepting the DHI intervention and completing at least one virtual visit. The percentage is calculated as the proportion of remote CR enrollments

^c^Total number of patients enrolling in the DHI intervention with completion. Percentage calculated as the proportion of DHI enrollment

### Functional status, cardiovascular risk factors, and patient-reported endpoints

Over 3 months, significant improvements in 6MWT distance (463 vs. 441 m in the post-intervention versus pre-intervention groups, respectively), average weekly step counts (39,983 vs. 30,616 steps per week) total cholesterol (136 vs. 148 mg/dL), LDL-C (71 vs. 81 mg/dL), systolic (125 vs 129 mmHg) and diastolic (76 vs. 79 mmHg) BP, BMI (31.1 vs. 31.8) and smoking rates (10% vs 15%; MD, -5%; 95% CI, -8% to -2%; *P* < 0.01) were seen in a post- vs. pre-intervention analysis among DHI participants with available data (Table 3). No significant improvements were seen in either high-density lipoprotein (HDL) cholesterol or glycosylated hemoglobin levels over baseline. Significant improvements were seen in self-reported functional status using the DASI (41 vs. 33) and depressive symptoms with the PHQ-9 (4.4 vs. 6.1) over baseline among DHI participants with available data. There were no adverse patient events reported.

**Table 3:** Changes in functional, cardiovascular, and patient-reported endpoints in DHI participants at 3 months

	**DHI Group**				
	**Baseline** Mean ± SD	**3-Month Follow Up** Mean ± SD	**Patients with Full Data** N	**Within-Group Difference** MD (95% CI)	***P***
6-min walk distance, meters	441 ± 133	463 ± 135	97	29 (10 to 49)	< 0.01
Weekly activity, steps	30,616 ± 23,008	39,983 ± 29,403	93	+ 9367 (1412 to 10,162)	0.01
Total cholesterol, mg/dL	148 ± 44	136 ± 35	146	-10 (-16 to -4)	< 0.01
LDL cholesterol, mg/dL	81 ± 39	71 ± 32	145	-11 (-17 to -5)	< 0.01
HDL cholesterol, mg/dL	40 ± 11	40 ± 10	146	0.5 (-0.7 to 1.6)	0.5
Glycated hemoglobin, %	6.7 ± 1.6	6.6 ± 1.5	132	0.1 (-0.1 to 0.5)	0.17
Systolic blood pressure, mmHg	129 ± 17	125 ± 18	211	-4 (-7 to -2)	0.01
Diastolic blood pressure, mmHg	79 ± 11	76 ± 11	211	-2 (-3.9 to 0.2)	0.03
BMI	31.8 ± 6.2	31 ± 6	213	-0.4 (-0.7 to -0.2)	< 0.01
Current smoker	15%	10%	214	-5% (-8% to -2%)	< 0.01
DASI	33 ± 16.4	41 ± 15.5	142	7 (5 to 9)	< 0.01
PHQ-9	6.1 ± 5.3	4.4 ± 5.2	141	-1.7 (-2.4 to -1.1)	< 0.01

### DHI engagement

The degree of DHI participants’ engagement with the various smartphone app features is summarized in Table 4. The most frequently used features were self-tracking of medication adherence, weight, step counts (via the wearable device), and BP, with at least 50% or more participants using each. The feature with the most extended average duration of use was the wearable fitness tracker (mean 80 ± 73 days), followed by BP (mean 54 ± 56 days) and weight (mean 47 ± 55 days) logging.

**Table 4:** Number of participants and duration of engagement with the Movn app

	**DHI Group** (*N* = 258)	
	**Participants Using** **App Feature**^a^ N (%)	**Duration** Mean days ± SD
Exercise session, self-reported	117 (45)	32 ± 41
Step counts, via the wearable device	130 (50)	80 ± 73
BP	129 (50)	54 ± 56
Weight	133 (52)	47 ± 55
Smoking	43 (17)	8 ± 29
Medication adherence	151 (59)	17 ± 37

^a^The number of unique participants using the feature at least once per day

## Discussion

In this pragmatic study of DHI-enhanced remote CR, we showed that implementation of a technology-enhanced CR remote CR program including a smartphone app, wearable fitness tracker, and coaching dashboard with live monitoring was feasible in a population of veterans within the VA healthcare system. Participants also showed improvements over baseline with nearly all health parameters measured, including 6-min walk distance, cardiovascular risk factors (e.g., LDL-C, systolic and diastolic BP, and BMI), health behaviors (e.g., weekly activity, smoking status), functional status, and depressive symptoms. By adopting technology, veterans were able to benefit from improved monitoring and increased access to their provider, which is particularly important given the high level of morbidity and access barriers they otherwise may face with multiple chronic conditions, rurality, and low socioeconomic status [29].

Digital health technologies are now used broadly across the consumer and healthcare delivery sectors [19, 30]. Integrating digital technologies into CR delivery has the potential to overcome many of the current challenges of traditional center-based programs while augmenting care delivery and expanding access [31]. Even among patients who participate in CR, there is potential to better support them with adopting sustained behavior change using digital technologies [31]. In a recent systematic review of 22 randomized controlled trials, smartphones and mobile devices (e.g., wearables) were the most commonly used digital technologies (65% of studies) [18]. Smartphone apps have been associated with improved patient outcomes when automated exercise recording, real-time feedback, and correctional goal-setting features are incorporated [32]. However, according to a recent Science Advisory from the American Heart Association, evidence supporting digital technologies in CR still points to significant gaps that must be addressed before broader implementation into routine practice [31].

Therefore, our study responds to this critical need for evidence by supporting the pragmatic implementation of patient-centered CR strategies that can be efficiently deployed in response to the rapidly shifting needs of patients and healthcare systems [33]. Given the abundance of controlled randomized trials and controlled studies already performed using DHIs with varying remote CR strategies across multiple populations [34–36], we decided to use a pragmatic open-label study design to evaluate the real-world impact of a DHI within a routine clinical setting to increase engagement through technology. This approach had several specific advantages in a veteran population, in whom few have access to traditional center-based CR, [37, 38] but otherwise do have access to digital tools for communication and monitoring [39, 40]. The VA has also invested significantly in digital health tools to offer remote care, and this program was in line with their larger strategic goals.

Although we did not present a control group due to limited data, our findings are in line with results from the literature on similar programs that health status improves in multiple domains after participation in structured exercise and risk reduction programs [41]. As with other lifestyle change programs, several measures that changed as a result of this program are not routinely addressed as a part of standard cardiology management such as depressed mood and sedentary behavior, which underscores its value [42]. Also, many potential benefits of the program were also difficult to quantify. For example, medication reminders, survey delivery, two-way messaging, and video education content likely enhanced the quality of care for these participants.

Successful widescale adoption of DHIs into the full spectrum of CR delivery, including remote programs, depends on several factors. First, although the state of evidence continues to expand and support using digital technologies in more vulnerable populations with studies such as ours, a more robust framework for comprehensive and equity-centered digital health in CR is needed [31]. Second, the recent expansion of artificial intelligence and “smart” wearable devices in cardiovascular care that provide more automated feedback to the patient beyond basic data collection presents an opportunity to integrate digital biomarkers into remote CR [43]. Third, the wide heterogeneity in the design and specific DHI components used in both research and clinical remote CR programs require guidance on standardization of data collection and reporting to ensure more consistency for outcome reporting and value-based assessments. Lastly, sustainable reimbursement models for DHIs and remote CR will be essential for effectively translating these new approaches into clinical care [44].

### Limitations

Our study has several limitations. All the participants were veterans and members of an integrated healthcare system consisting primarily of males, which limits its generalizability. Nonetheless, the cohort was racially diverse, as nearly half were Black. We also had limited power to analyze differences based on referral diagnosis, including ischemic (acute MI, CAD, chronic angina) and non-ischemic (heart failure, valve surgery) heart disease. Another limitation was the lack of a control group, which was in part due to limited data and the pragmatic nature of this study. As such, we focused primarily on factors like overall enrollment, which is impressive considering that normally less than 10% of eligible Veterans participate in CR. Nonetheless, veterans who were not comfortable with technology, who are often sicker and older, were not enrolled in the DHI. The coronavirus pandemic resulted in many canceled in-person data collection visits and missing data, highlighted in Table 3. Nonetheless, we were able to retrieve some information from the electronic health record and outside records when available to increase our sample size. The Movn app itself also helped with certain data collection such as steps, vitals, and patient-reported outcomes during the pandemic.

## Conclusions

In conclusion, we found that remote CR with DHI was feasible in a VA hospital setting which serves many high-risk patients with low socioeconomic status, and also where many such studies are challenging to implement because of VA-specific privacy requirements. Health status of participants improved which was in line with other similar programs. Despite the challenges of implementing technology in such an environment, many veterans were able to engage on a daily basis, and completion rates were high. More research is needed to understand the potential role of this program at other centers and its potential impact on long-term outcomes such as mortality.

## Data Availability

The datasets used and/or analyzed during the current study are available from the corresponding author on reasonable request.

## Abbreviations

CR: Cardiac rehabilitation
DHI: Digital health intervention
VA: Veterans Affairs
VHA: Veterans Health Administration
6MWT: 6-Minute walk test
RPE: Rate of perceived exertion
BP: Blood pressure
LDL-C: Low density lipoprotein-cholesterol
BMI: Body mass index
PROMs: Patient reported outcome measures
DASI: Duke Activity Status Index
PHQ-9: Patient Health Questionnaire-9
